# Cold-Inducible RNA-Binding Protein Is an Important Mediator of Alcohol-Induced Brain Inflammation

**DOI:** 10.1371/journal.pone.0079430

**Published:** 2013-11-01

**Authors:** Salil R. Rajayer, Asha Jacob, Weng-Lang Yang, Mian Zhou, Wayne Chaung, Ping Wang

**Affiliations:** 1 Department of Surgery, Hofstra North Shore-LIJ School of Medicine, Manhasset, New York, United States of America; 2 Center for Translational Research, The Feinstein Institute for Medical Research, Manhasset, New York, United States of America; University of Cincinnati, United States of America

## Abstract

Binge drinking has been associated with cerebral dysfunction. Ethanol induced microglial activation initiates an inflammatory process that causes upregulation of proinflammatory cytokines which in turn creates neuronal inflammation and damage. However, the molecular mechanism is not fully understood. We postulate that cold-inducible RNA-binding protein (CIRP), a novel proinflammatory molecule, can contribute to alcohol-induced neuroinflammation. To test this theory male wild-type (WT) mice were exposed to alcohol at concentrations consistent to binge drinking and blood and brain tissues were collected. At 5 h after alcohol, a significant increase of 53% in the brain of CIRP mRNA was observed and its expression remained elevated at 10 h and 15 h. Brain CIRP protein levels were increased by 184% at 10 h and remained high at 15 h. We then exposed male WT and CIRP knockout (CIRP^−/−^) mice to alcohol, and blood and brain tissues were collected at 15 h post-alcohol infusion. Serum levels of tissue injury markers (AST, ALT and LDH) were significantly elevated in alcohol-exposed WT mice while they were less increased in the CIRP^−/−^ mice. Brain TNF-α mRNA and protein expressions along with IL-1β protein levels were significantly increased in WT mice, which was not seen in the CIRP^−/−^ mice. In cultured BV2 cells (mouse microglia), ethanol at 100 mM showed an increase of CIRP mRNA by 274% and 408% at 24 h and 48 h respectively. Corresponding increases in TNF-α and IL-1β were also observed. CIRP protein levels were markedly increased in the medium, suggesting that CIRP was secreted by the BV2 cells. From this we conclude that alcohol exposure activates microglia to produce and secrete CIRP and possibly induce pro-inflammatory response and thereby causing neuroinflammation. CIRP could be a novel mediator of alcohol-induced brain inflammation.

## Introduction

In the United States, over fifty percent of the adult population consumes alcohol on a regular basis [1]. In 2006, the CDC had estimated the economic cost of excessive drinking as about $220 billion [2]. Binge drinking was responsible for >70% of these costs. Binge drinking is defined by the National Institute on Alcohol Abuse and Alcoholism (NIAAA) as ≥4 drinks for a woman and ≥5 drinks for a man on a single occasion. Typically, these results of alcohol concentrations are in the range of the legal intoxication limit (i.e., a blood alcohol level of 80 mg/dL). Thus, the cost incurred while treating any of the acute conditions related with intoxication can be fully attributed to binge drinking [2]. A number of studies have shown that binge drinking leads to impairment of cognitive function and several mechanisms have been proposed to account for this brain dysfunction [3]–[7]. Among these, central nervous system (CNS) inflammation is one of the proposed explanations for alcohol induced brain dysfunction.

The primary effectors of neuroinflammation are microglia cells, which are the resident macrophages of the brain. They form a significant portion of the CNS cell population, constituting approximately 20% of the total glial cell population and are almost as numerous as neurons [8]. At rest, microglia seem to perform various homeostatic functions, involving themselves in synaptic plasticity and neurotransmission [9], [10]. The activation of microglia in response to stimuli can initially be protective for neurons [11]–[14]. However, over activation of these cells from various conditions can lead to inflammatory products that may eventually cause neuronal destruction which is observed in various CNS pathologies [15]–[21]. Excessive alcohol consumption has also been shown to promote inflammation in the CNS [22]–[24]. These findings in recent years have established a link between alcohol and neuroinflammation. However, the molecular factors involved in these processes have not yet been comprehensively delineated.

CIRP is a 172-aa molecule belonging to the family of cold shock proteins which are involved in binding single stranded nucleic acids. It consists of one amino-terminal consensus sequence RNA-binding domain and one carboxyl-terminal glycine-rich domain. CIRP was discovered in 1997 by Nishiyama et al [25] and is constitutively expressed in a wide variety of tissues in low amounts and plays a role in cellular processes such as transcription, translation and DNA recombination. It acts as an RNA chaperone to facilitate translation [26]. It is also highly expressed in certain conditions like hypothermia, hypoxia, and ultraviolet irradiation [27]–[29]. Recently, it has also been found to play an important role in the circadian rhythm of living cells [30]. Interestingly, Saito et al exposed rats to alcohol for 15 months and analyzed their dorsal hippocampus for gene expression changes using cDNA microarrays. They found a two fold increase in mRNA of a gene termed AA818118 which is similar to CIRP [31]. Our recent study showed that healthy animals injected with recombinant murine CIRP (rmCIRP) were found to have significantly elevated serum levels of liver enzymes (AST and ALT) and cytokine TNF-α indicating CIRP as a potent proinflammatory agent [32]. Based on these findings, we postulated that CIRP may play a role in the alcohol induced proinflammatory cascade in the brain.

## Materials and Methods

### Mouse model of acute binge alcohol

Male C57BL/6 mice (20–25 g, Taconic, Albany, NY) were used as Wild-type (WT) mice in all experiments. CIRP^−/−^ mice on a background of C57BL/6 were a gift from Dr Jun Fujita (Kyoto University, Japan) and are bred in our animal facility. Upon acquisition, WT mice were allowed to acclimate to the environment in our facility for 5–7 days before the experiment. All mice were housed in cages of five members each and subjected to 12 h light/dark cycles. Each type of mouse (WT or CIRP^−/−^) was randomly divided into 2 groups: Saline (Sham) or Alcohol. Anesthesia was induced with 2.5% inhalational isoflurane. The right internal jugular vein was exposed with a 0.5-cm neck incision and a PE-10 catheter was inserted via a venotomy. The catheter was sutured in place and connected via a harness (SAI Infusion Technologies, Libertyville, IL) to an infusion pump (KD Scientific, Holliston, MA). The harness allowed for free movement of the mice in the cage while receiving a continuous infusion. Alcohol group animals received an initial bolus of 43.7 mg/25 g ethanol followed by 7.5 mg/25 g/h for 15 h bringing the total amount to 156 mg/25 g of ethanol. At the end of 15 h, animals were anesthetized and blood and brain tissue samples were harvested and stored at −80°C. For determining time-course changes, only WT animals were used. They were anesthetized and cannulated identical to the procedure as described above. Alcohol group animals received the same initial bolus of ethanol followed by continuous infusion for 5, 10 or 15 h. At the end of each corresponding time period, animals were anesthetized and blood and brain tissue samples were harvested. Serum alcohol levels were measured by a commercially available alcohol kit (Pointe Scientific, MI).

All experiments were performed in strict accordance with the guidelines for the use of experimental animals by the National Institute of Health and were approved by the Institutional Animal Care and Use Committee of The Feinstein Institute of Medical Research. All surgery was performed under 2.5% isoflurane anesthesia and all efforts were made to minimize suffering.

### Measurement of serum levels of injury markers

Blood samples were centrifuged at 2,000 g for 15 min to collect serum. The serum levels of alcohol and activity of organ injury markers aspartate aminotransferase (AST), alanine aminotransferase (ALT) and lactate dehydrogenase (LDH) were measured by using assay kits from Pointe Scientific (Canton, MI).

### Measurement of tissue cytokine levels

Tissue lysate protein concentrations were determined using Bio-Rad DC protein assay kit (BIO-RAD, Hercules, CA). TNF-α and IL-1β levels were determined in the samples by ELISA kits from BD Biosciences (San Jose, CA) as per the manufacturer's protocols and represented as pg/mg protein.

### Culture of microglial cells

The immortalized murine BV2 cell line (BV2 cells), which exhibits both the phenotypic and functional properties of reactive microglia cells [33], [34] were obtained as a kind gift from Dr Philippe Marambaud (Feinstein Institute for Medical Research, NY) and cultured in Dulbecco's Modified Eagle's Medium (DMEM, Invitrogen) supplemented with 10% fetal bovine serum (FBS), 1% penicillin-streptomycin, and 1% glutamine, as previously described [35], [36].

### Alcohol stimulation of BV2 cells

BV2 cells in their third passage were plated in triplicates at 2×10^6^ per well (six well plates) in complete DMEM. Plated cells were then incubated at 37°C, 5% CO_2_ overnight to enable attachment. The next day the culture medium was changed to OPTI-MEM (reduced serum medium, Life technologies, Grand Island, NY). One hour later, these plates were exposed to either sterile phosphate buffered saline (PBS) or endotoxin free 99.9% Ethanol (Sigma-Aldrich, St. Louis, MO) at concentrations of 50 mM and 100 mM. These concentrations were chosen because they were in the range of blood alcohol levels found among alcoholics [37]. The plates were incubated for 24 h or 48 h at 37°C, 5% CO_2_. Cell lysates and supernatants were collected at the end of the corresponding time-period and stored in −80°C before being subjected to analysis. Three separate BV2 microglia-alcohol stimulation experiments were performed.

### Reverse transcriptase-polymerase chain reaction (RT-PCR) analysis

Total RNA was extracted from brain tissues and cell lysates using Trizol (Invitrogen, Carlsbad, CA). cDNA was reverse-transcribed from 2 µg of total RNA using Oligo (dT)_12–18_ primer (Life Technologies, Grand Island, NY) and murine leukemia virus reverse transcriptase (Applied Biosystems, Foster City, CA). A PCR reaction was done in 25 µl of final volume containing 0.08 µmol of each forward and reverse primer, cDNA, and 12.5 µl SYBR Green PCR Master Mix (Applied Biosystems, Foster City, CA). Amplification was conducted in an A lied Biosystems 7300 real-time PCR machine under the temperature of 50°C for 2 min, 95°C for 10 min and 45 cycles of 95°C for 15 seconds and 60°C for 1 min. Relative expression of each mRNA was calculated using ^ΔΔ^Ct threshold model. Mouse β-actin was used for normalization. Relative expression of mRNA was represented as fold change in comparison to sham/control levels. The primers used are listed in Table 1.

**Table 1:** Real-Time PCR primers used in this study.

Gene	GenBank #	Forward (5′-3′)	Reverse (5′-3′)
**CIRP**	NM_031168	CCAGAGGAGACTTCACAG	CAGAATTGCCATTGCACAAC
**TNF-α**	NM_007705	AGGACTCAGCTTCGACACCA	CGTCCACAGACTTCCCATTC
**IL-1β**	NM_008361	CAGGATGAGGACATGAGCACC	CTCTGCAGACTCAAACTCCAC
**β-Actin**	NM_007393	CGTGAAAAGATGACCCAGATCA	TGGTACGACCAGAGGCATACAG

### Western blotting for CIRP protein

#### Brain tissue and cell lysate

Brain tissue and cell lysates were homogenized in lysis buffer (10 mM Tris-HCl pH 7.5, 120 mM NaCl 1% NP-40, 1% sodium deoxycholate, and 0.1% sodium dodecyl sulfate) with protease inhibitor (Roche Diagnostics, Indianapolis, IN) by gentle sonication (Sonic Dismembranator 100, Fisher Scientific, Pittsburgh, PA). Protein concentrations were determined using Bio-Rad DC protein assay kit (Bio-Rad, Hercules, CA). Total lysate was fractioned on Bis-Tris gels (4–12%) and transferred to nitrocellulose membranes. The membranes were then blocked with 5% milk in 0.2 X PBS and then incubated with anti-CIRP (Proteintech Group, Chicago, IL) or β-actin primary antibodies (Santa Cruz Biotechnology, Santa Cruz, CA). After washing, the membranes were incubated with a fluorescently-labeled secondary antibody (LI-COR, Lincoln, NE). Bands were detected with the Odyssey FC Dual-Mode Imaging system 2800 (LI-COR, Lincoln, NE) and the band intensity measured using the NIH Image J densitometric software.

### Supernatant

Cell supernatant proteins were precipitated using the DOC-TCA method. The precipitate was then subjected to Western blotting for CIRP as described above. The band densities were equalized using Ponceau S Solution (0.1% Ponceau S (w/v) in 5% acetic acid (v/v), Sigma-Aldrich, St. Louis, MO).

### Statistical analysis

All data are expressed as mean ± SE and compared by one-way analysis of variance (ANOVA) and the Student-Newman-Keuls test. Differences in values were considered significant if p<0.05.

## Results

### Serum alcohol levels after the continuous infusion

The blood alcohol level was measured as 66 mg/dL at 5 h after the initiation of the continuous alcohol infusion, and significantly increased to 132 mg/dL at 10 h and finally at the end of the full infusion was about 156 mg/dL (Table 2). The level of blood alcohol at 15 h is consistent with reported levels of binge drinking habits [38] and it was about twice the legal intoxication limit of 80 mg/dL.

**Table 2:** Serum alcohol levels after the continuous infusion.

	Serum Alcohol Level (mg/dL)
**Sham**	Not Detectable
**5 h**	66±4.6
**10 h**	132±2.7*
**15 h**	156±23.0*

Data presented as means ± SE (n = 3–6/group) and compared by one-way ANOVA and SNK method.

* p<0.05 vs. 5 h group.

### Acute binge alcohol increased brain CIRP level in a time dependent manner

**Figure 1:**
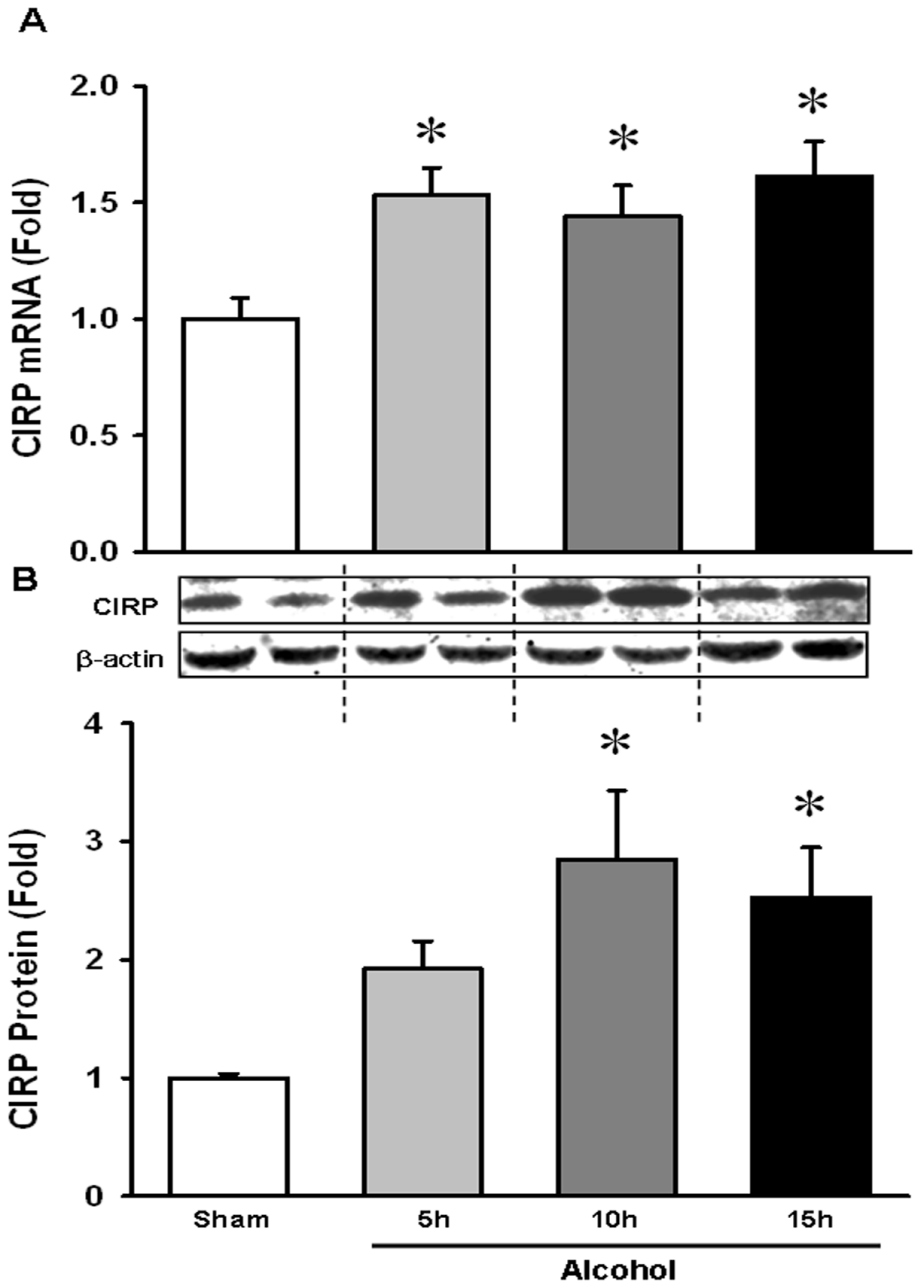
Alcohol increased brain CIRP level in a time dependent manner. WT mice were infused intravenously with alcohol as described in Materials and Methods. Whole brain tissue from WT mice was collected at 5, 10 and 15(**A**) CIRP mRNA expression was determined by real time RT-PCR analysis and expression levels were normalized to β-actin. (**B**) Tissue lysates were collected and analyzed for CIRP by Western blotting. Blots were scanned and quantified by densitometry. Band intensity of CIRP was normalized to the corresponding band intensity of β-actin. The ratio of the control group is designated as 1 for comparison. Data presented as means ± SE (n = 3–4/group) and compared by one-way ANOVA and SNK method; *p<0.05 vs. Sham.

Prior studies have shown that CIRP is highly expressed in stress conditions such as hypothermia, hypoxia and UV irradiation [27], [28]. Our recent study showed that CIRP is induced and released into the circulation in hemorrhagic shock and sepsis [32]. Based on these findings, we examined whether CIRP is increased in the brain in WT mice exposed to alcohol. Towards that end, we evaluated the mRNA and protein levels of WT mice exposed to alcohol for 5, 10 and 15 h. We found that there was a significant 53% induction of CIRP mRNA level at 5 h and it remained elevated until 15 h (Fig. 1A). A corresponding significant increase by 184% in protein level was observed at 10 h and remained high at 15 h as compared to unexposed mice (Fig. 1B). This suggests that acute binge alcohol induces CIRP expression in the brain.

### Clinical markers of organ injury were attenuated in CIRP^−/−^ mice following alcohol exposure

**Figure 2:**
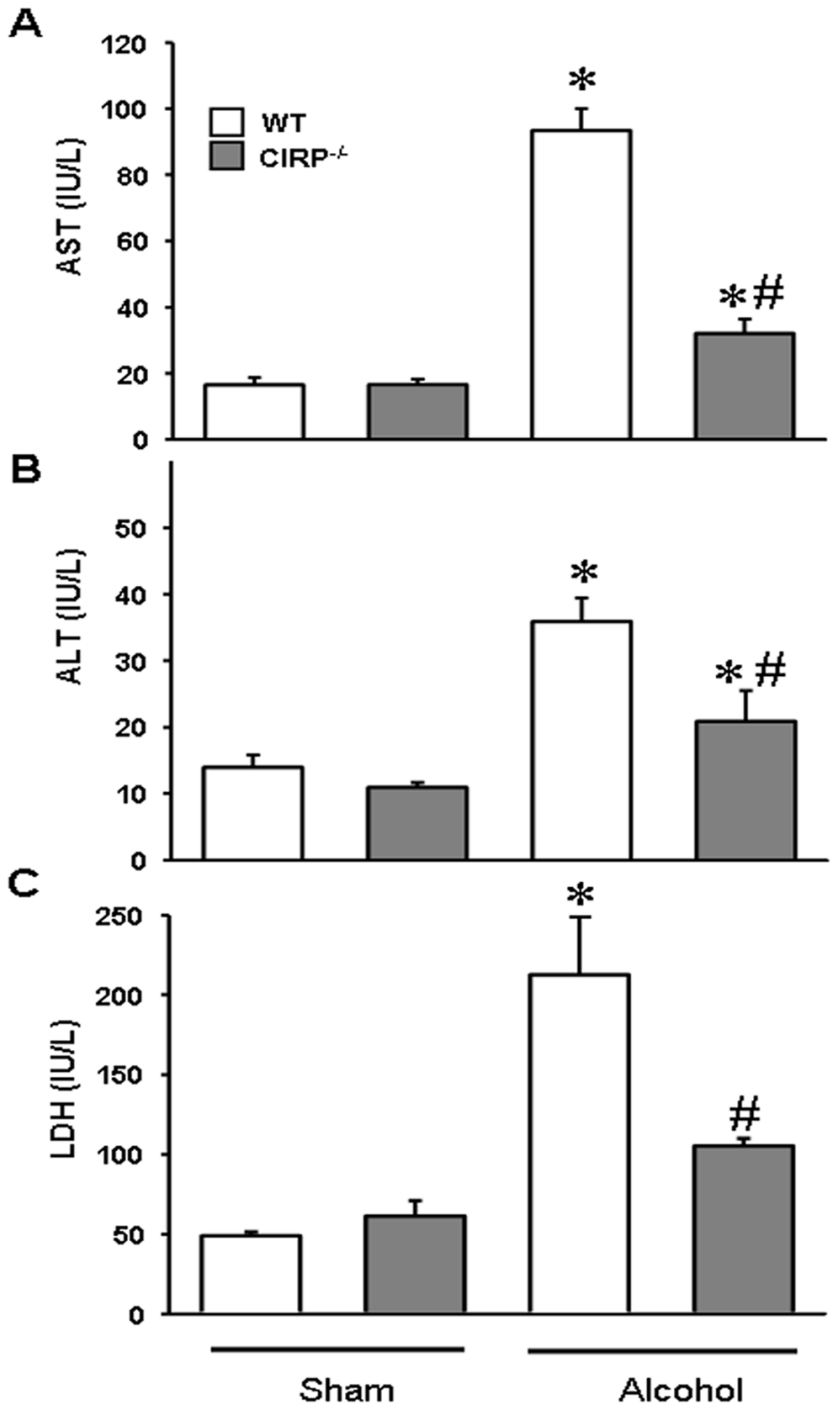
Clinical markers of organ injury were improved in CIRP^−/−^ following alcohol exposure. WT and CIRP^−/−^ mice were infused intravenously with alcohol for 15 h. Serum was collected and analyzed for AST (**A**), ALT (**B**) and LDH (**C**) using standardized assays. Data are presented as means ± SE (n = 5/group) and compared by one-way ANOVA and SNK method; *p<0.05 vs. respective Sham and #p<0.05 vs. WT alcohol group.

To delineate the importance of the increased CIRP expression in the brain, we evaluated the effects of alcohol on mice in the absence of the CIRP protein. WT and CIRP^−/−^ mice were infused intravenously with alcohol for 15 h as previously described. As systemic markers of organ injury, serum levels of enzymes AST, ALT and LDH were measured. Alcohol infusion in the WT animals caused significant increases of 467%, 157% and 321% in serum AST, ALT and LDH respectively (Figs. 2A–C). In the CIRP^−/−^ mice, these values were decreased by 66%, 42% and 47% respectively, when compared to the alcohol-exposed WT mice, indicating a significantly decreased degree of tissue injury (Figs. 2A–C). We also measured AST, ALT and LDH at 5 h and 10 h after alcohol infusion in the WT mice. All three parameters increased with time after the start of the alcohol infusion (Table 3).

**Table 3:** Time-course changes in tissue injury markers and TNF-α after acute alcohol.

	Sham	5 h	10 h	15 h
**Serum AST**	13.6±1.1	55.5±6.7*	48.0±16.1*	97.5±6.9* **^#^** †
**Serum ALT**	9.7±1.1	15.7±1.7	23.3±4.6* #	31.5±1.9* **^#^** †
**Serum LDH**	50.4±1.7	154.7±17.3*	123.0±27.4	221.0±36.4*

Data presented as means ± SE (n = 3–5/group) and compared by one-way ANOVA and SNK method.

* p<0.05 vs. Sham,

# p<0.05 vs. 5 h,

† p<0.05 vs.10 h groups.

### Brain pro-inflammatory cytokines TNF-α and IL-1β were diminished in CIRP^−/−^ following alcohol exposure

**Figure 3:**
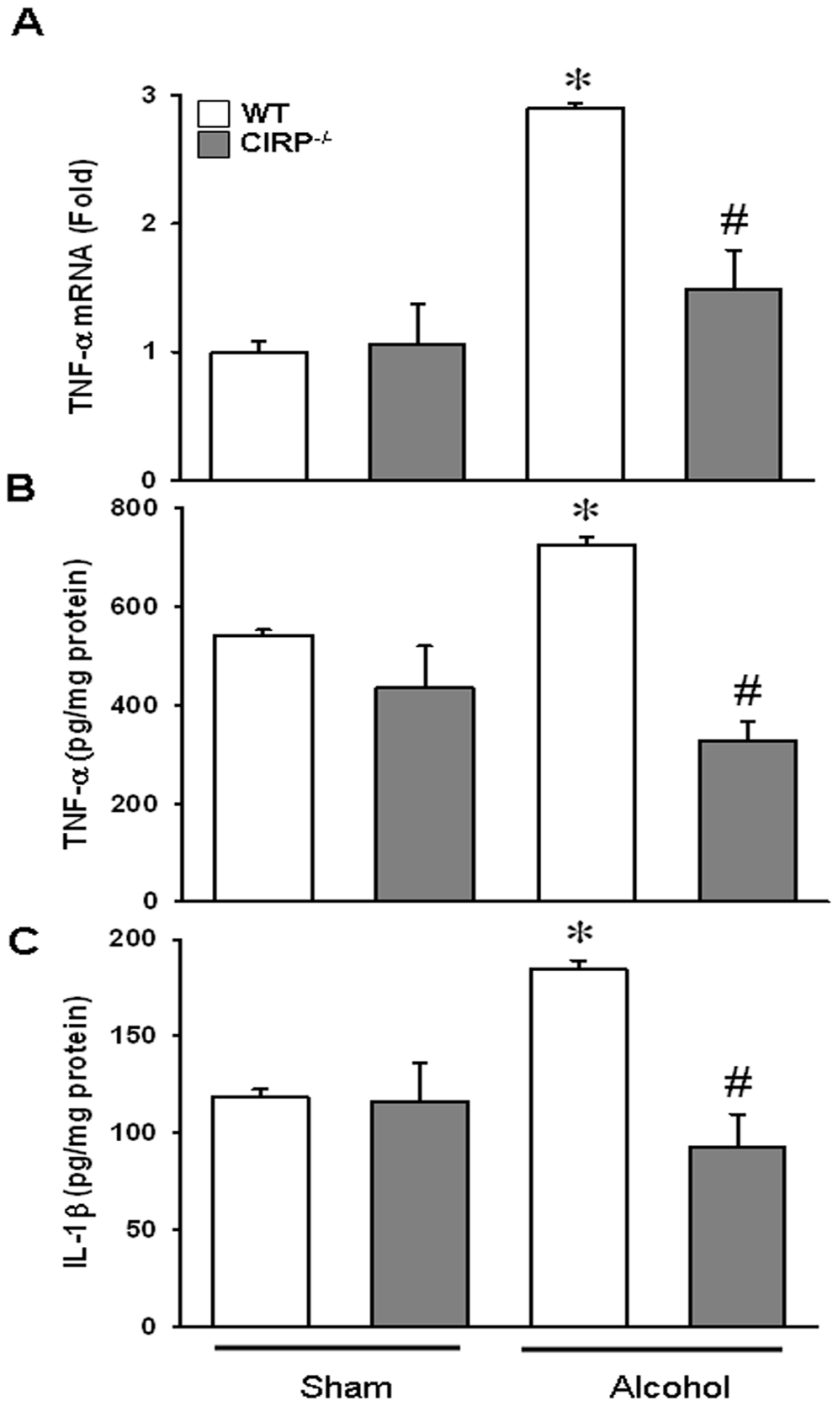
Brain proinflammatory cytokines TNF-αand IL-1β were attenuated in CIRP^−/−^ following alcohol exposure. Whole brain samples from WT and CIRP^−/−^ mice were collected at 15 h after alcohol and analyzed. TNF-α mRNA expression (**A**) was determined by real time RT-PCR analysis and expression levels were normalized to β-actin. Brain protein cytokine levels of TNF-α (**B**) and IL- 1β (**C**) were determined by subjecting cell lysates to standardized mouse ELISA (BD Biosciences). Data are presented as means ± SE (n = 3–5/group) and compared by one-way ANOVA and SNK method;*p<0.05 vs. Sham, #p<0.05 vs. WT alcohol groups.

Next, it was explored if the increase in CIRP was associated with an increase in the cytokine levels in the brain following alcohol infusion. We found that in the WT animals, the 15 h alcohol infusion significantly increased the brain TNF-α mRNA expression and protein levels by 127% and 34%, respectively. In the CIRP^−/−^ mice however, TNF-α mRNA and protein levels were attenuated by 78% and 55% respectively, as compared to alcohol exposed WT mice (Figs. 3A and 3B). Likewise, IL-1β protein levels were increased by 58% in alcohol exposed WT mice and these levels were decreased by 50% in the CIRP^−/−^ mice (Fig. 3C). This suggests that in the absence of CIRP, the microglial activation and by corollary, the proinflammatory effects created by alcohol exposure are significantly attenuated in mice. Serum levels of the cytokines TNF-α and IL-1β were also measured but were undetectable.

### Alcohol upregulated gene expression of CIRP and cytokines TNF- α and IL-1β in BV2 cells

**Figure 4:**
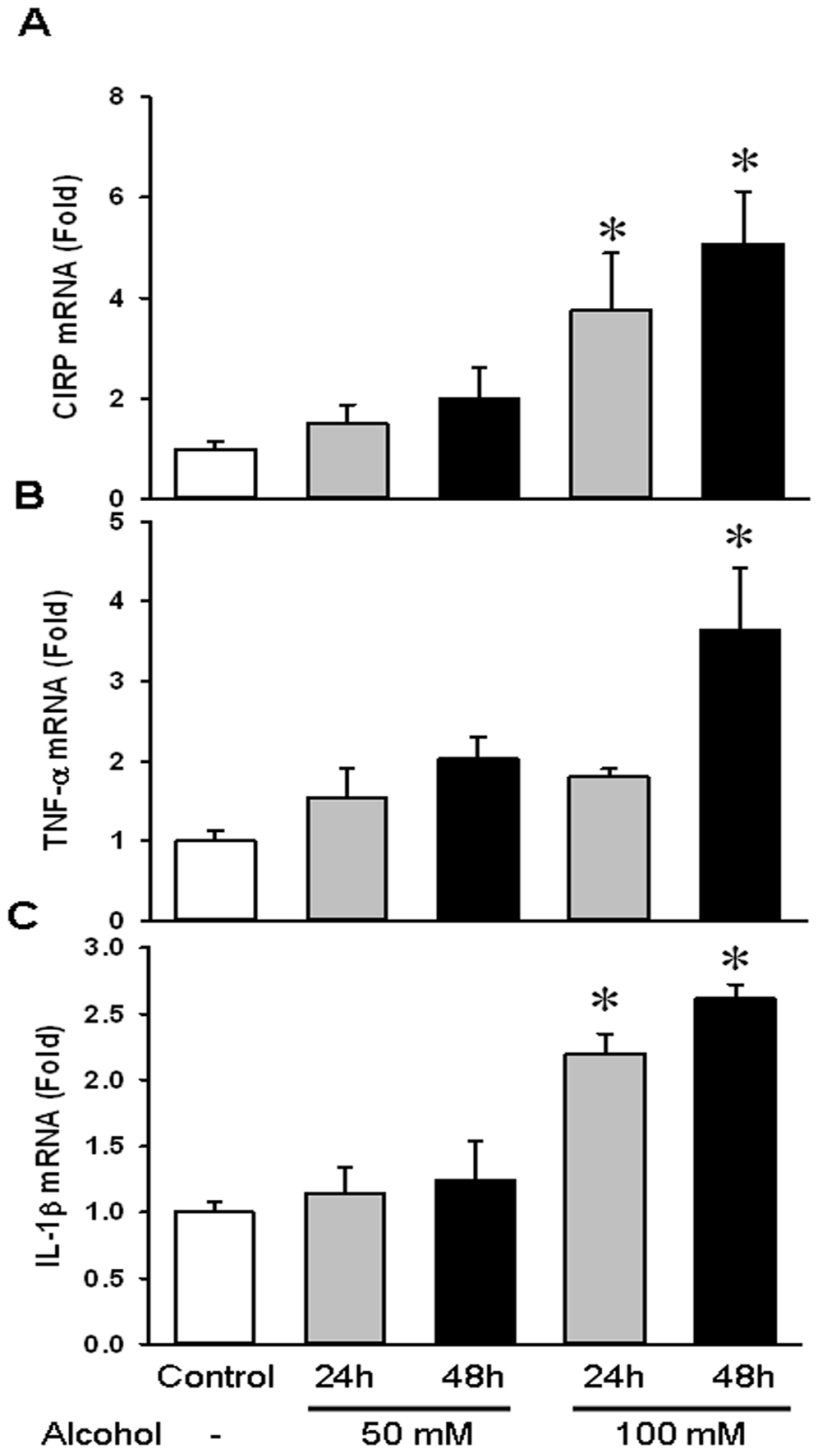
Alcohol upregulated gene expression of CIRP and cytokines TNF-α and IL-1β in BV2 cells. BV2 Cells were exposed to ethanol at a concentration of 50(**A**), TNF-α (**B**), and IL-1β (**C**) mRNA expressions were determined by real time RT-PCR analysis and expression levels were normalized to β-actin. The ratio of the control group is designated as 1 for comparison. Data are presented as means ± SE (n = 3–5/group) and compared by one-way ANOVA and SNK method; *p<0.05 vs. Control.

Alcohol exposure has been shown to recruit microglia and cause their activation. As mentioned earlier, microglial activation consists of a characteristic pattern of cellular responses including morphological and functional changes. These involve cell proliferation, expression of immunomolecules, recruitment to the injured region and the release of cytotoxic and/or inflammatory mediators [39]. In our study, BV2 cells were exposed to ethanol at a concentration of 50 mM and 100 mM over 24 h and 48 h. We found that gene expression of CIRP in cells treated with 100 mM ethanol was increased by 274% and 400% at 24 h and 48 h respectively (Fig. 4A). We also examined the mRNA levels of TNF-α and IL-1β. While TNF-α levels were only significantly elevated by 262% at 48 h at 100 mM ethanol dose, IL-1β levels were significantly upregulated by 120% and 161% at 24 h and 48 h respectively, at the same dose (Figs. 4B–C). Thus, acute alcohol caused microglial activation leading to increased gene expression of CIRP and increases in TNF-α and IL-1β.

### CIRP protein was secreted into the cell culture medium following alcohol exposure

**Figure 5:**
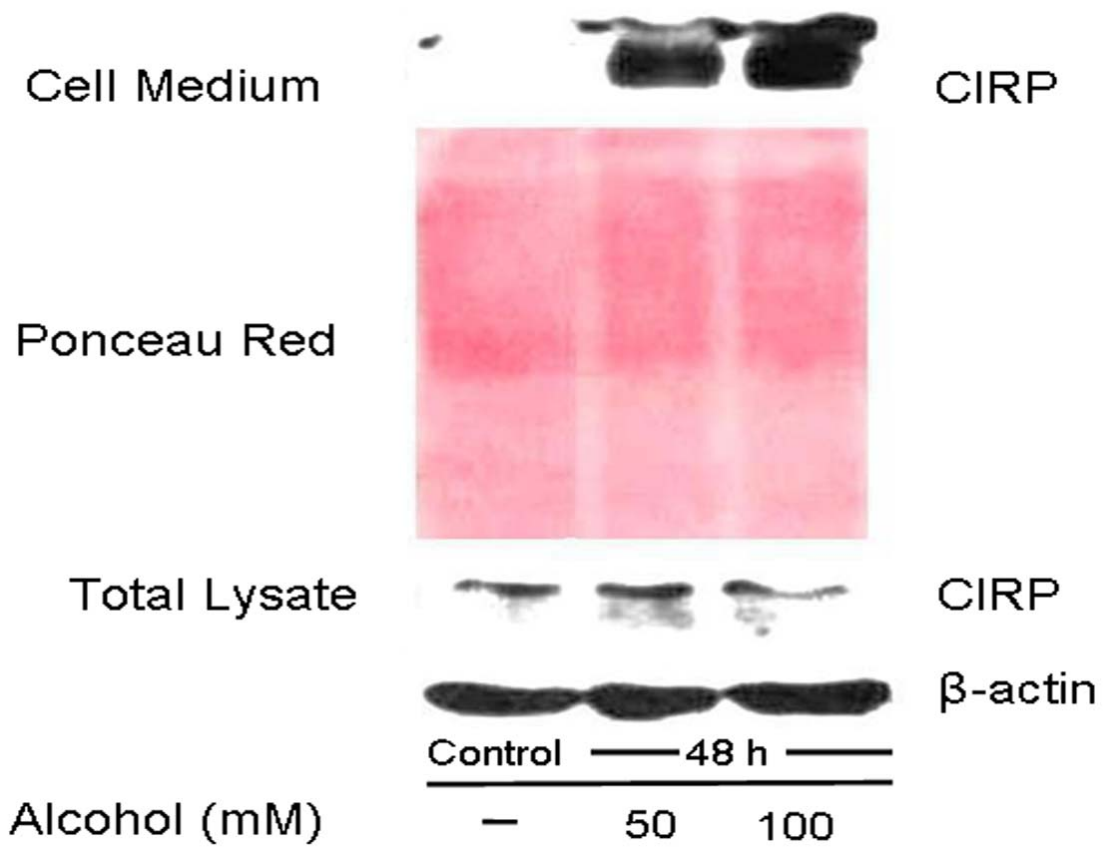
CIRP protein was secreted into the cell culture medium following alcohol exposure. BV2 Cells were exposed to ethanol at a concentration of 50

To determine whether CIRP could be secreted into the cell medium, we collected the supernatants and cell lysates separately from BV2 cells treated with 50 and 100 mM alcohol for 48 h and analyzed them by Western blotting. We found that while CIRP protein was decreased in the total cell lysate at 48 h, it was markedly increased in the cell culture medium at both concentrations tested (Fig. 5). Thus, alcohol causes microglial activation, leading to the secretion of CIRP protein into the medium.

## Discussion

Binge drinking has become the most popular form of alcohol abuse, especially among the youth [40]. The damaging effects of alcohol abuse on the developing brain in young people, i.e. retardation of intellectual function, rational decision making and emotional maturation have been well documented [41]. The earlier paradigm that moderate alcohol consumption is neuroprotective has now been reversed [42]. Alcohol induced immune activation in the brain has been widely considered responsible for its deleterious effects [43].

In our current study, we first showed that binge alcohol intoxication increased brain levels of CIRP, both at the transcriptional and translational level. This increase was correlated with increases in the serum organ injury markers and proinflammatory cytokine levels in the brain. We then showed that in the absence of CIRP, i.e. in CIRP^−/−^mice, the serum organ injury markers and brain cytokine levels were markedly diminished suggesting a critical role of CIRP in alcohol-induced neuroinflammation. Finally, similar to what was observed from the whole brain tissue, we demonstrated that alcohol exposure in BV2 cells, a mouse brain microglia cell line, increased both CIRP mRNA and protein levels indicating that the cell type responsible for CIRP upregulation could be brain microglial cells. We further showed *in vitro* that alcohol exposure caused CIRP to be secreted into the medium. Thus, we implemented both *in vivo* and *in vitro* approaches and identified CIRP as a novel mediator of alcohol-induced neuroinflammation in mice. To our knowledge, this is the first report that CIRP as a contributor to alcohol-induced neuroinflammation.

Central nervous system inflammation is one of the proposed explanations for alcohol induced brain dysfunction. The primary effectors of neuroinflammation are microglia. The activation of microglia in response to stimuli can be triggered either by families of pattern recognition receptors such as the Toll-Like receptors or due to the cessation of neuroprotective signals from receptor interactions [11]. Activation of microglia might initially be protective for neurons. When they are exposed to invading pathogens, neuronal debris, or proinflammatory cytokines and chemokines, microglia rapidly change to an activated state [12]. They play a part in regulating the regeneration of neurons and remodeling of the brain by producing a variety of cytotoxic as well as neuroprotective molecules [13]. In lesions where there is breakdown of the blood-brain barrier such as cerebral ischemia, brain abscesses and traumatic injuries causing, microglial activation along with further macrophage recruitment and debris clearance takes place [14]. However, overactivation of these cells in various conditions leads to inflammatory products that eventually may cause neuronal destruction as observed in various CNS pathologies. Microglial cells play an active part in degenerative CNS diseases such as Alzheimer's [20] and Parkinson's diseases [16]. A role for neuroinflammation via microglial activation is also seen in Amyotrophic Lateral Sclerosis[15]. In fact, neuroinflammation has even been seen with Down's Syndrome [21], which may predispose these patients to undergo neurodegeneration. In most neurological autoimmune diseases such as multiple sclerosis, microglia induced phagocytosis is the pathological hallmark [17]. In the human acquired immunodeficiency syndrome (AIDS) virus infected macrophages probably introduce the virus to the CNS and in concert with microglia are involved in the pathophysiology of the AIDS dementia complex [18]. Recently, light has been shed on the role of microglial activation and neuroinflammation in neurodevelopmental disorders such as autism [19]. It is clear that neuroinflammation via microglial activation plays a major role in the pathology of CNS disease and dysfunction.

Excessive alcohol consumption also promote inflammation in the CNS [44]. Ethanol increases expression of brain pro-inflammatory genes through activation of the transcription factor, NF-κB [22] and transgenic mice lacking TLR4 are protected against ethanol-induced upregulation of pro-inflammatory genes, glial activation, and neurotoxicity [24]. Ethanol induces microglia activation by stimulating TLR4 response and that the inflammatory response induced by ethanol is completely abrogated in microglia of TLR4^−/−^ mice [45].

In the present study, we show that alcohol increased CIRP mRNA and protein expressions in the mouse brain. Without CIRP, alcohol-induced serum levels of organ injury markers, and brain TNF-α and IL-1β levels were markedly diminished. Furthermore, we demonstrated that alcohol increased CIRP mRNA and protein expressions in mouse microglial cells and caused its release into the medium. Our recent study showed that CIRP interacts with TLR4 and facilitates inflammatory responses in macrophages [32]. We therefore suggest that alcohol stimulates the production and secretion of CIRP from microglial cells in the brain, CIRP then interacts with TLR-4 on the microglial cells, activating them to release proinflammatory cytokines. Thus, CIRP plays a role in alcohol-induced neuroinflammation. In summary, as a marker for alcohol-induced neuroinflammation, or as a target for therapy in alcohol use disorders, CIRP may play an important role in the future.
